# Supplementary figures and images for: New insights into intranuclear inclusions in thyroid carcinoma: Association with autophagy and with BRAFV600E mutation
Source: PLoS One. 2019 Dec 16;14(12):e0226199. doi: 10.1371/journal.pone.0226199 (PMC6913918; doi:10.1371/journal.pone.0226199)

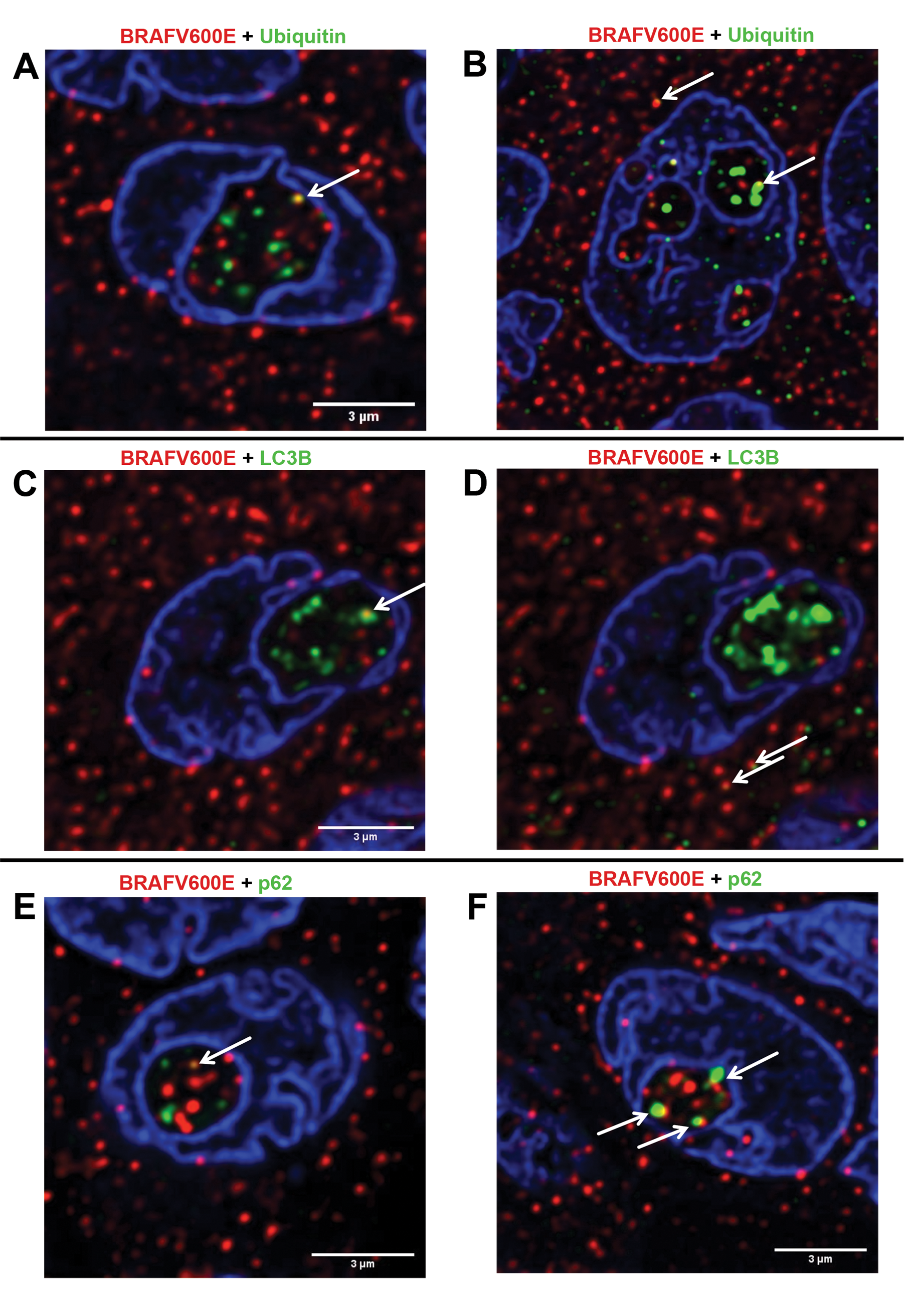

Supplement: S1 Fig — (A-B) BRAFV600E/ubiquitin double IF labelling (A) The image demonstrates co-localization of mutant BRAF (red) with ubiquitin (green) within the inclusions (arrow) proved by the merged color yellow with lack of co-localization in the cytoplasm (B) Another nucleus with inclusions containing an accumulation of ubiquitin is seen with co-localization of BRAFV600E/ubiquitin both within the intranuclear inclusion (NI) and in the cytoplasm (arrows). Both the merged color yellow and the immunoreactivity for ubiquitin are stronger in the NI than in the cytoplasm; only weak distribution of ubiquitin is seen in the cytoplasm. (C-D) After z-stack analysis of the same nucleus BRAFV600E/LC3B co-localizations were found on two different z-planes (C) Co-localization of BRAFV600E (red) with LC3B (green) in the NI (arrow) but not in the cytoplasm is shown in this single z-plane image. (D) Few co-localizations of mutant BRAF with LC3B are seen in the cytoplasm (arrows) with no co-localization within the same NI on the other z-plane. Immunostaining for LC3B was stronger in the NI than in the cytoplasm demonstrating an accumulation of LC3B within the NI. (E-F) BRAFV600E/p62 double IF labelling reveals co-localization of BRAFV600E (red) with p62 (green) in two inclusions proven by the merged color yellow (arrows) with lack of co-localization in the cytoplasm. (TIF) [file pone.0226199.s001.tif]
