# Supplementary material for: New insights into intranuclear inclusions in thyroid carcinoma: Association with autophagy and with BRAFV600E mutation
Source: PLoS One. 2019 Dec 16;14(12):e0226199. doi: 10.1371/journal.pone.0226199 (PMC6913918; doi:10.1371/journal.pone.0226199)
Supplement: S1 Table — (PDF) [file pone.0226199.s002.pdf]

Supporting information

S1 Table. Immunohistochemistry antibodies and staining protocols

Antibody	Company	Clonality	Clone	HIER	Dilution	Incubation	Second. Antibody	Company	Dilution	Incubation	Detection	Company	Incubation	DAB	Hematoxylin
Ubiquitin	Agilent Technologies	Rb. poly.		pH 9.0; 20 min. WB 98°C	/1000	30min. RT	-	-	-	-	Zytomed polymer HRP	Zytomed	Kit	2x5min.	5min.
p62	Santa Cruz	Ms. mono.	D-3	pH 9.0; 20 min. WB 98°C	/1000	30min. RT	-	-	-	-	Zytomed polymer HRP	Zytomed	Kit	2x5min.	5min.
LC3B	Nano Tools	Ms. mono.	5F10	pH 9.0; 20 min. WB 98°C	/3000	30min. RT	-	-	-	-	Zytomed polymer HRP	Zytomed	Kit	2x5min.	5min.
Cathepsin B	Santa Cruz	Rb. poly.		pH 9.0; 20 min. WB 98°C	/500	30min. RT	-	-	-	-	Zytomed polymer HRP	Zytomed	Kit	2x5min.	5min.
Cathepsin D	Santa Cruz	Gt. poly.		pH 9.0; 20 min. WB 98°C	/6000	60min. RT	Rb Anti Gt	Chemicon AP106	/1000	30min.RT	Zytomed polymer HRP	Zytomed	Kit	2x5min.	5min.
Lamin AC	Cell Signaling	Rb. poly		pH 9.0; 20 min. WB 98°C	/25	60min. RT					Cell Signaling boost Rb polymer HRP	Cell Signaling	Kit	2x5min	5min.
BRAFV600E	abcam	Ms. mono	VE1	pH 9.0; 20 min. WB 98°C	/400	30min. RT					Zytomed polymer AP	Zytomed	Kit	Permanent red 2x10min.	5min.

Antibodies and staining protocols are shown in detail. From each FFPE tissue 3-to-5 µm-thick sections were cut, dewaxed and pre-treated for further processing in an automated staining device (Dako Autostainer, Dako Glostrup Denkmark). Abbreviations: Rb: rabbit; Ms: mouse; Gt: goat; poly: polyclonal; mono: monoclonal; WB: water bath; HIER: heat-induced epitope retrieval.
