# Supplementary material for: New insights into intranuclear inclusions in thyroid carcinoma: Association with autophagy and with BRAFV600E mutation
Source: PLoS One. 2019 Dec 16;14(12):e0226199. doi: 10.1371/journal.pone.0226199 (PMC6913918; doi:10.1371/journal.pone.0226199)
Supplement: S2 Table — (PDF) [file pone.0226199.s003.pdf]

Supporting information

S2 Table. NGS study: Panel of analyzed genes and exons

gene	exons
APC	all
ARID1A	all
ARID1B	all
ARID2	all
AXIN1	all
BRAF	11, 15
CSNK1A1	all
CSNK1D	all
CSNK1E	all
CSNK1G1	all
CTNNB1	all
DICER1	all
FZD3	all
FZD6	all
FZD7	all
GSK3A	all
GSK3B	all
KRAS	2-4
LRP5	all
LRP6	all
NRAS	2-4
RPS6KA3	all
SMAD4	all
WNT3a	all
WNT4	all
WNT5a	all
