# Supplementary material for: New insights into intranuclear inclusions in thyroid carcinoma: Association with autophagy and with BRAFV600E mutation
Source: PLoS One. 2019 Dec 16;14(12):e0226199. doi: 10.1371/journal.pone.0226199 (PMC6913918; doi:10.1371/journal.pone.0226199)
Supplement: S3 Table — (PDF) [file pone.0226199.s004.pdf]

Supporting information

S3 Table. 3D-Imaging of the inclusions: Antibodies used for double-immunofluorescence and staining conditions

anti-	manufacturer	order number	host	dilution	Incubation conditions
Lamin AC	Abcam	Ab193904	rabbit monoclonal	1:50	overnight 4°C

B: Fluorochrome labeling

Lamin AC	Donkey F(ab')2 antiRabbit<br>Invitrogen A21206<br>(Alexa Fluor 488)	1:100, room temperature<br>60min.
