# Supplementary material for: New insights into intranuclear inclusions in thyroid carcinoma: Association with autophagy and with BRAFV600E mutation
Source: PLoS One. 2019 Dec 16;14(12):e0226199. doi: 10.1371/journal.pone.0226199 (PMC6913918; doi:10.1371/journal.pone.0226199)
Supplement: S4 Table — (PDF) [file pone.0226199.s005.pdf]

Supporting information

S4 Table. Double-labeling immunofluorescence microscopy: LC3B/ubiquitin, p62/ ubiquitin and LC3B/p62

Double-labeling immunofluorescence microscopy for LC3B and ubiquitin

A: Primary Antibodies

anti-	manufacturer	order number	host	dilution	Incubation conditions
LC3B	Cell Signaling	3868	mono rabbit<br>D11	1:20	room temperature<br>30min. 2x
Ubiquitin	Novus	NB300-130	Mono Mouse IgG1<br>Ubi-1	1:1000	room temperature,<br>30min. 2x

B: Fluorochrome labeling

LC3B	Goat Anti Rabbit<br>Dianova 111-166-045<br>Cy3	1:100; room temperature,<br>30min. 2x
Ubiquitin	Goat Anti Mouse<br>Invitrogen A21121<br>Alexa Fluor 488	1:100; room temperature,<br>30min. 2x

Double-labeling immunofluorescence microscopy for p62 and ubiquitin

A: Primary Antibodies

anti-	manufacturer	order number	host	dilution	Incubation conditions
p62	Enzo	BML PW9860	Poly Rabbit	1:500	room temperature, 30min.
Ubiquitin	Novus	NB300-130	Mono Mouse IgG1 Ubi1	1:1000	room temperature, 30min.

B: Fluorochrome labeling

p62	Goat Anti Rabbit Dianova 111-166-045 Cy3	1:800; room temperature, 30min.
Ubiquitin	Goat Anti Mouse Invitrogen A21121 Alexa Fluor 488	1:100; room temperature, 30min.

Double-labeling immunofluorescence microscopy for LC3B and p62

A: Primary Antibodies

anti-	manufacturer	order number	host	dilution	Incubation conditions
LC3B	Cell Signaling	3868	Mono Rabbit<br>D11	1:20	room temperature<br>30min. 2x
p62	Santa Cruz	Sc-28359	Mono Mouse IgG1<br>D2	1:20	room temperature,<br>30min. 2x

B: Fluorochrome labeling

LC3B	Goat Anti Rabbit<br>Dianova 111-166-045<br>Cy3	1:800; room temperature ,<br>30min. 2x
p62	Goat anti Mouse<br>Invitrogen A21121<br>AlexaFluor 488	1:100; room temperature,<br>30min., 2x
