# Supplementary material for: New insights into intranuclear inclusions in thyroid carcinoma: Association with autophagy and with BRAFV600E mutation
Source: PLoS One. 2019 Dec 16;14(12):e0226199. doi: 10.1371/journal.pone.0226199 (PMC6913918; doi:10.1371/journal.pone.0226199)
Supplement: S5 Table — (PDF) [file pone.0226199.s006.pdf]

Supporting information

S5 Table. Double-labeling immunofluorescence microscopy: BRAFV600E/LC3B, BRAFV600E/p62 and BRAFV600E/ubiquitin

Double-labeling immunofluorescence microscopy for BRAFV600E and LC3B

A: Primary Antibodies

**anti-**	**manufacturer**	**order number**	**host**	**dilution**	**Incubation conditions**
BRAFV600E	abcam	ab228461	Mono Mouse IgG2a<br>VE1	1:100	room temperature,<br>60min., 2x
LC3B	Cell Signaling	#3868	Mono Rabbit<br>D11	1:100	room temperature,<br>60min.,2x

B: Fluorochrome labeling

BRAFV600E	Vecta Fluor Excel<br>Vector DK-2594<br>DyLight 594	ready to use, room temperature,<br>30min., 2x
LC3B	Donkey Anti Rabbit<br>Invitrogen A21206<br>Alexa Fluor 488	1:100, room temperature<br>60min., 2x

Double-labeling immunofluorescence microscopy for BRAFV600E and p62

A: Primary Antibodies

anti-	manufacturer	order number	host	dilution	Incubation conditions
BRAFV6000E	abcam	ab228461	Mono Mouse IgG2a<br>VE1	1:100	room temperature,<br>60min. 2x
p62	Enzo	BML PW9860	Poly Rabbit	1:250	room temperature,<br>60min.,1x

B: Fluorochrome labeling

BRAFV600E	Vecta Fluor Excel<br>Vector DK-2594<br>DyLight 594	ready to use, room temperature,<br>30min., 2x
p62	Donkey Anti Rabbit<br>Invitrogen A21206<br>Alexa Fluor 488	1:100, room temperature,<br>60min.,1x

Double-labeling immunofluorescence microscopy for BRAFV600E and ubiquitin

A: Primary Antibodies

anti-	manufacturer	order number	host	dilution	Incubation conditions
BRAFV600E	abcam	ab228461	Mono Mouse IgG2a<br>VE1	1:100	room temperature,<br>60min.,2x
Ubiquitin	Dako	Z0458	Poly Rabbit	1:500	room temperature,<br>60min.,1x

B: Fluorochrome labeling

BRAFV600E	Vecta Fluor Excel<br>Vector DK-2594<br>DyLight 594	ready to use, room temperature,<br>30min., 2x
Ubiquitin	Donkey Anti Rabbit<br>Invitrogen A21206<br>Alexa Fluor 488	1:100, room temperature<br>60min.,1x
