# Supplementary material for: New insights into intranuclear inclusions in thyroid carcinoma: Association with autophagy and with BRAFV600E mutation
Source: PLoS One. 2019 Dec 16;14(12):e0226199. doi: 10.1371/journal.pone.0226199 (PMC6913918; doi:10.1371/journal.pone.0226199)
Supplement: S6 Table — (PDF) [file pone.0226199.s007.pdf]

Supporting information S6 Table. NGS study: Mutations in the analyzed genes of the thyroid carcinoma cohort[a]

Gene	AA Mutation Cosmic_v70	n cases (%)
APC	*S62F*	1 /107 (0.9 %)
APC	*R499**	1 /107 (0.9 %)
APC	*W699**	1 /107 (0.9 %)
APC	*G1106R*	1 /107 (0.9 %)
APC	*A1107V*	1 /107 (0.9 %)
APC	*Q1260**	1 /107 (0.9 %)
APC	*E1306K*	1 /107 (0.9 %)
APC	*S1327L*	1 /107 (0.9 %)
APC	*S1355F*	1 /107 (0.9 %)
APC	*G1365D*	1 /107 (0.9 %)
APC	*P1634L*	2 /107 (1.8 %)
ARID1A	*Q502**	1 /107 (0.9 %)
ARID1A	*G778R*	1 /107 (0.9 %)
ARID1A	*Q1409**	1 /107 (0.9 %)
ARID1A	*R1722**	1 /107 (0.9 %)
ARID1A	*E2035K*	1 /107 (0.9 %)
ARID1A	*G2177R*	2 /107 (1.8 %)
ARID1B	*G510R, G568R*	1 /107 (0.9 %)
ARID1B	*P985L*	1 /107 (0.9 %)
ARID1B	*V1498I, V1516I*	1 /107 (0.9 %)
ARID1B	*R1534K, R1552K*	3 /107 (2.7 %)
ARID1B	*G1804S, G1822S*	2 /107 (0.9 %)
ARID2	*N309D*	1 /107 (0.9 %)
ARID2	*Q476**	1 /107 (0.9 %)
ARID2	*Q1403**	1 /107 (0.9 %)
ARID2	*R1754**	1 /107 (0.9 %)
AXIN1	*A76T*	1 /107 (0.9 %)
AXIN1	*W85**	2 /107 (1.8 %)
AXIN1	*E87K*	2 /107 (1.8 %)
AXIN1	*H90Y*	2 /107 (1.8 %)
AXIN1	*D113N*	1 /107 (0.9 %)
AXIN1	*Q186**	1 /107 (0.9 %)
AXIN1	*S228L*	1 /107 (0.9 %)
AXIN1	*W247**	1 /107 (0.9 %)
AXIN1	*P314L*	1 /107 (0.9 %)

Gene	AA Mutation Cosmic_v70	n cases (%)
BRAF	*V600E*	14 /107 (13.1 %)
CSNK1A1	-	0 /107 (0 %)
CSNK1D	-	0 /107 (0 %)
CSNK1E	*G243D*	1 /107 (0.9 %)
CSNK1E	*P403S*	1 /107 (0.9 %)
CSNK1G1	-	0 /107 (0 %)
CTNNB1	*E9K*	1 /107 (0.9 %)
CTNNB1	*E15K*	1 /107 (0.9 %)
CTNNB1	*H24Y*	1 /107 (0.9 %)
CTNNB1	*H36Y*	1 /107 (0.9 %)
CTNNB1	*S37F*	1 /107 (0.9 %)
DICER1	*Y560C*	1 /107 (0.9 %)
DICER1	*R935K*	1 /107 (0.9 %)
FZD3	-	0 /107 (0 %)
FZD6	-	0 /107 (0 %)
FZD7	*D322N*	1 /107 (0.9 %)
GSK3A	-	0 /107 (0 %)
GSK3B	*R180W*	1 /107 (0.9 %)
KRAS	*V14I*	1 /107 (0.9 %)
KRAS	*E49K*	1 /107 (0.9 %)
LRP5	*G1110D*	1 /107 (0.9 %)
LRP6	*R1184**	1 /107 (0.9 %)
NRAS	*Q61R*	5 /107 (4.7 %)
RPS6KA3	*V527I*	1 /107 (0.9 %)
SMAD4	-	0 /107 (0 %)
WNT3a	-	0 /107 (0 %)
WNT4	-	0 /107 (0 %)
WNT5a	-	0 /107 (0 %)

[a]**:** All mutations found in Cosmic with the prevalence of minimum 5 % are listed; mutations listed additionally in the Clinvar database are marked in green.
